# New fat-derived products for treating skin-induced lesions of scleroderma in nude mice

**DOI:** 10.1186/scrt528

**Published:** 2014-12-17

**Authors:** Nicolas Serratrice, Laurie Bruzzese, Jérémy Magalon, Julie Véran, Laurent Giraudo, Houssein Aboudou, Djaffar Ould-Ali, Pierre Sébastien Nguyen, Olivier Bausset, Aurélie Daumas, Dominique Casanova, Brigitte Granel, Lucile Andrac-Meyer, Florence Sabatier, Guy Magalon

**Affiliations:** Department of Plastic and Reconstructive Surgery, CHU La Conception (AP-HM), 147, Boulevard Baille, 13005 Marseille, France; Department of Anatomopathology, CHU Nord (AP-HM), Chemin des Bourrely, 13015 Marseille, France; Culture and Therapy Unit, CHU La Conception (AP-HM), 147, Boulevard Baille, 13005 Marseille, France; CIC Biotherapy, INSERM CBT510, 147, Boulevard Baille, 13005 Marseille, France; Department of Internal Medicine, CHU La Timone (AP-HM), 264, Rue Saint-Pierre, 13005 Marseille, France; Department of Internal Medicine, CHU Nord (AP-HM), Chemin des Bourrely, 13015 Marseille, France; Vascular Research Center of Marseille, INSERM UMRS-1076, Aix-Marseilles University, 27, Boulevard Jean Moulin, 13385 Marseille, France

## Abstract

**Introduction:**

Scleroderma is characterized by cutaneous manifestations that mainly affect the hands, arms and face. As of today, there is no treatment for fibrotic skin lesions of scleroderma. Previously we generated and validated a model of scleroderma-like skin sclerosis in nude mice, appropriate to inject human derived products. We showed that the subcutaneous injection of micro-fat (MF), purified and injected using small caliber cannulas, have anti-fibrotic and pro-angiogenic effects and appears more suitable for the treatment of skin lesions of scleroderma compared to the gold standard (Coleman’s technique or macro-fat). Here we compared the long-term efficacy of micro-fat “enriched” with other therapeutic products including the stromal vascular fraction (SVF) of fat and platelet-rich plasma (PRP) from blood in our murine model of scleroderma.

**Methods:**

We used 72 nude mice in this study. We formed six experimental groups: Macro-fat, MF, SVF, PRP, MF + SVF, MF + PRP. This project has three phases: i) Induction of skin sclerosis by daily subcutaneous injections of bleomycin (BLM) for 4 weeks in nude mice; ii) Purification and injection of the different cell therapy products; iii) Histological analyses done 8 weeks post-injections.

**Results:**

MF + SVF and MF + PRP significantly reversed dermal and epidermal sclerosis (*P* <0.01). Macro-fat, SVF, PRP only corrected the dermal sclerosis (*P* <0.05). Epidermal sclerosis was reduced in treatments containing MF (*P* <0.01). MF was more stable. Products containing the SVF were associated with a significant increase of the local vascularization (*P* <0.01).

**Conclusions:**

All tested substances were effective in treating skin-induced lesions of scleroderma with different levels of fibrosis and vascular improvement; MF derived products are more stable and SVF demonstrated better pro-angiogenic effects. The observed efficacy of this combination of products in the animal model provides a rationale for potential clinical applications to treat human disease.

## Introduction

Over the years, new approaches to fat transfer – also referred to as lipoinjection or lipofilling – have been explored, studied extensively and refined. In 1995, Dr Sydney Coleman developed a simple fat transfer procedure [1], which has rapidly become the reference technique in reconstructive and cosmetic surgery. Briefly, the procedure consists of a gentle and atraumatic manual aspiration of the subcutaneous fat tissue, usually in the abdomen or trochanters, using a 3 mm diameter suction cannula mounted on a 10 cm^3^ syringe. After a short centrifugation at 1,200 g for 3 minutes to remove the blood, oil and tissue residuals, adipose tissue is purified and directly reinjected subcutaneously with a 17 G cannula. Owing to its autologous origin, adipose tissue injection appears safe.

Experience has shown that fat transfers may have a true regenerative effect in addition to the volumizing effect [2]. Mature adipocytes represent only 40 to 60% of the cells of the adipose tissue. The fat also contains the stromal vascular fraction (SVF), within which mesenchymal stem cells are located. Numerous studies have characterized the nature of the SVF stem cells. These mesenchymal-like stem cells are able to develop *in vitro* muscle, bone, cartilage, neural, epithelial, macrophage or even hepatocyte phenotypes [3–12]. SVF also contains a large number of fibroblasts characterized by membrane markers CD34^+^ and CD90^+^. SVF is capable of secreting collagen and matrix metalloproteinases and of organizing extracellular matrix [13]. In addition, SVF cells behave as endothelial progenitors with strong angiogenic potential [14–18]. Paracrine and autocrine effects of reinjected SVF cells play a major role in the dermal tissue quality, recruiting nearby stem cells, and stimulating also surrounding differentiated cells [19, 20]. Several recent studies have shown that fat prepared according to the Coleman procedure mixed with the SVF increases graft survival compared with fat alone [21–23]. This new concept of fat enrichment or supercharged fat offers new therapeutic perspectives.

However, adipose tissue obtained using the Coleman procedure is relatively thick: it forms 1 mm diameter fat filaments, limiting injections into subcutaneous space – subdermal injections being relatively tricky. As a result, an innovative technique of microinjection for adipose tissue called microinjection has been developed in the laboratory. The sampling and reinjection methods have been modified to obtain micro-fat (MF), more fluid, which can be injected through smaller needles [24]. The technique involves removal and purification of MF by gentle suction with a 1 mm diameter micro-blunt cannula with multiple holes mounted on a 10 cm^3^ syringe and by a short centrifugation at 1,200 g/minute for 2 minutes. MF is composed of micro-adipocyte lobules (around 500 μm) that can be reinjected through a 21 G cannula (0.8 mm).

Scleroderma is a chronic systemic autoimmune disease primarily of the skin (‘derma’) characterized by fibrosis or hardening (‘sclero’), vascular alterations, and autoantibodies [25, 26]. Various cutaneous manifestations are possible in scleroderma (morphea, *en coup de sabre*). At present, there is no consensus treatment for skin lesions of scleroderma and injection of adipose tissue could represent a viable option. We previously compared the efficacy of injection of fat prepared according to the Coleman procedure versus MF in a nude mice model of scleroderma [27]. We evidenced histologically that the MF injection was more efficient than fat injection in reducing bleomycin (BLM)-induced injury, as shown by reduction of dermis thickness (*P* <0.05 with Coleman’s technique and *P* <0.01 with the technique of microinjection) and improved vascularization of the deep dermis and subcutis (*P* <0.01). Otherwise, these results are consistent with previously reported clinical data for localized scleroderma [28, 29] as well as other skin lesions [30]. Hence, fat microinjection appeared more suitable for injection or subdermal fibrotic tissue. However, no study has investigated whether enriching MF with SVF could optimize the therapeutic potential of this procedure in scleroderma.

The use of platelet-rich plasma (PRP) represents another attractive option for the development of combined biological therapy. Owing to its capacity to deliver various growth factors, PRP has been investigated as a regenerative treatment in various conditions including nerve injury, tendinitis, osteoarthritis, cardiac muscle injury, bone repair and regeneration, plastic surgery, maxillofacial and oral surgery [31–35]. Various *in vitro* studies indicate that PRP may have beneficial impact on the regenerative potential of mesenchymal stem cells [36]. However, very few studies have examined the use of a fat mixture (prepared according to Coleman) and PRP. In 2012, Gentile and colleagues reported that a fat + PRP mix increases graft survival compared with fat alone, thus maintaining a volumizing effect in breast reconstruction [22]. No study has been conducted on a MF + PRP mix.

This study was conducted to evaluate and compare efficacy of these different cell therapy products – macro-fat (Coleman’s procedure), MF, SVF, PRP, MF + SVF and MF + PRP – in the nude mice model of skin-induced lesions of scleroderma. A beneficial effect of fat grafting on the sclerotic skin should have potential clinical applications to treat human disease or other soft tissue defects.

## Methods

### Animals

The National Animal Care and Ethics Committee approved the care of mice and the experimental procedures (#00506.02). Based on the results of previous work, we determined that six mice/group would be sufficient to show a statistically significant difference between the different cell therapy products. Seventy-two pathogen-free female nude mice (6 weeks old, weight ~20 g) were purchased from Charles River Laboratories (L’arbresle, France). Forty-eight nude mice were used for the experiment, and 24 mice as controls. The experiment was conducted in the new A2 animal facility of the Centre de Formation et de Recherches Expérimentales Médico-Chirurgicales of the Faculty of Medecine of Marseilles. Mice were acclimated for 3 weeks before any experimental procedures. No mice died prematurely during experimentation.

### Bleomycin treatment

BLM treatment was specially adapted from Yamamoto’s original protocol for nude mice [27, 37]. Briefly, BLM (Sanofi-Aventis, Paris, France) was dissolved in 0.9% sodium chloride saline solution at a concentration of 300 μg/ml. Forty-eight mice (9 weeks old) were injected subcutaneously into three different shaved parts known to have the same skin thickness – the interscapular region and both flanks – with a volume of 100 μl (30 μg) BLM, using a 26 G cannula, 7 days per week for 4 consecutive weeks. Mice were housed for one additional week without any treatment for recovery before injections of the different cell therapy products under general anesthesia.

In parallel, 12 mice were injected in the same sites with 100 μl of 0.9% sodium chloride saline solution for 4 weeks and were housed for one additional week without further treatment. Twelve supplementary naive mice for all treatments were used as negative controls.

Skin sclerosis was evaluated at 5 and 13 weeks by histological analyses on 12 mice randomly selected from the 48 BLM-treated mice, the 12 sodium chloride-treated mice and the 12 control mice (*n* = 6 for each time point).

### Experimental groups

Six experimental groups of six nude mice were randomly constituted (Table 1).

**Table 1:** **Experimental conditions and composition for each cell therapy product**

Group	Cell therapy products	Compositions
1	Coleman (gold standard)	0.5 cm^3^
2	MF	0.5 cm^3^
3	SVF	0.5 cm^3^ RL + 0.0114 cm^3^ SVF
4	PRP	0.25 cm^3^ PPP + 0.25 cm^3^ PRP
5	MF + SVF	0.5 cm^3^ MF + 0.0114 cm^3^ SVF
6	MF + PRP	0.25 cm^3^ MF + 0.25 cm^3^ PRP

Final volume/mouse = 0.5 cm^3^. MF, micro-fat; PPP, platelet-poor plasma; PRP, platelet-rich plasma; RL, Ringer’s lactate; SVF, stromal vascular fraction.

### Cell therapy products

A single, healthy volunteer donor was included after informed consent. All of the cell therapy products were purified and injected on the same day. This study did not need ethical approval for the use of human-derived products for research, because fat was considered surgical waste and no genetic research was carried out. Fat (according to Coleman’s method) and MF were collected from the lateral abdomen/flank areas during an abdominal dermolipectomy as described above. During the intervention, peripheral whole blood was taken at the elbow. The donor had no relevant diseases and was free of any drugs known to affect platelet functions for 7 days before the study. Standard serological tests were performed on whole blood for the safety of manipulations. All procedures were conducted under a class A microbiological safety facility located in the Culture and Therapy Unit at La Conception Hospital (Assistance Publique – Hôpitaux de Marseille), Marseilles, France.

#### Coleman’s technique

Fat was aspirated gently with a manual and nontraumatic technique, using Coleman’s blunt cannula (length 15 cm, internal diameter 2.42 mm; that is, 11 G) mounted with a 10 cm^3^ syringe.

#### Stromal vascular fraction

Fat (220 cm^3^) was aspirated according to Coleman’s technique. SVF was purified with the Celution® system (Cytori Therapeutics, San Diego, California, USA) following the recommended instructions. This system has been the subject of numerous publications in the areas of myocardial ischemia [38–41].

#### Micro-fat

MF (18 cm^3^) was aspirated with a cannula (length 15 cm, internal diameter 1 mm; that is, 14 G) mounted with a 10 cm^3^ Luer Lock syringe. MF was purified after a short centrifugation (1,200 × *g* during 3 minutes) with a microcentrifuge (Medilite®, Ref. 448; Thermo Scientific, Waltham, Massachusetts, USA) to eliminate oily and bloody residues.

#### Platelet-rich plasma

PRP was produced according to a previously described methodology [42]. Briefly, 68 cm^3^ whole blood were collected in eight 10 cm^3^ tubes (Vacuette®, Ref. 455001; Greiner Bio-One, Monroe, North Carolina, USA) with 1.5 cm^3^ adenosin citrate dextrose-acid solution (ACD-A®, Ref. BDB8651; Fenwal Inc., Portland, Oregon, USA), making 80 cm^3^. Another tube, coated with the ethylenediamine tetraacetic acid anticoagulant, was used to determine the platelet number and concentration with an automatic cell counter (ADVIA® 2120; Siemens Diagnostic Solutions, Malvern, Pennsylvania, USA). A first spin at 130 × *g* during 15 minutes and a second spin at 250 × *g* during 15 minutes in eight 11 cm^3^ conic tubes (NUNC®, Ref. 56423; Thermo Scientific, Waltham, Massachusetts, USA) were performed in a standard laboratory microcentrifuge (Medilite®, Ref. 448; Thermo Scientific). Supernatant or platelet-poor plasma (PPP) was removed by gentle aspiration (approximately 1 cm^3^/tube). PRP pellets were resuspended in the residual PPP and pooled. At final measurement, we obtained a total of 8.5 cm^3^ concentrated PRP. An approximately 250 μl sample was used to determine the PRP final formulation with an automatic cell counter (ADVIA® 2120; Siemens Diagnostic Solutions).

#### Mixes

The MF + SVF and MF + PRP compositions are reported in Table 1.

For each product, about 100 μl were injected into pediatric blood culture bottles (BacT/ALERT® device; bioMérieux, Craponne, France). Under these conditions, we obtained controlled and optimized cell therapy products. All products were packaged in 1 cm^3^ syringes for subsequent use. Ringer’s lactate was used to resuspend SVF and PPP for PRP.

### Injections

Under general volatile anesthesia (isoflurane (Forène®, Baxter France, Maurepas, France), concentration 2.5 to 3%, flow 400 to 600 ml/minute), a 1 mm incision was made on the external side of each thigh in view to introduce a 21 G blunt cannula for the MF, SVF, PRP, MF + SVF and MF + PRP injections. Macro-fat was injected using the classical Coleman’s procedure. One flank was injected with cell therapy products, whereas the other side was injected with the suitable resuspension product (Ringer’s lactate for SVF, and PPP for PRP) and served as the injection control. One-half of the mice in each experimental group received cell therapy products on the left and resuspension products on the right, and the other half received the contrary. The injection volume was 0.5 cm^3^ for all cell therapy products. Details of the different injected products are presented in Table 1. The total experimental duration was 8 weeks (2 months or 56 days); that is, mice were sacrificed at 22 weeks of age.

### Histological examination

Blinded histological analyses were conducted by a skin pathologist. A semiquantitative evaluation was used to evaluate cell therapy product viability during skin removal (0, no trace of the product; +, trace of the product; ++, cell therapy product still present). Skins were fixed in 10% formalin solution and embedded in paraffin. Then 5 μm thick sections were stained with hematoxylin and eosin, Masson’s trichrome, and orcein stainings to evaluate histopathological changes and to detect collagen fibers in tissues, and with toluidine blue staining to identify mast cells. Sections were examined using a light microscope (Axiophot; Zeiss, Oberkochen, Germany) and photographed with a digital camera. Epidermal and dermal thicknesses were measured quantitatively. Distances were calculated using GraphPad software (GraphPad, La Jolla, California, USA). Six measures were made and means were compared for each experimental condition. A semiquantitative evaluation was performed to evaluate vascularization (0, capillaries present in normal number and morphology; +, increased number of congested capillaries; ++, numerous congested capillaries).

### Statistical analyses

Statistical analysis was performed on Statview software (Statview, Cary, North Carolina, USA). Results are expressed as mean ± standard deviation. Significance testing was performed using analyses of variance. *P* <0.05 was considered significant.

## Results

### Pathological and clinical manifestations of the skin-induced model of scleroderma

**Figure 1:**
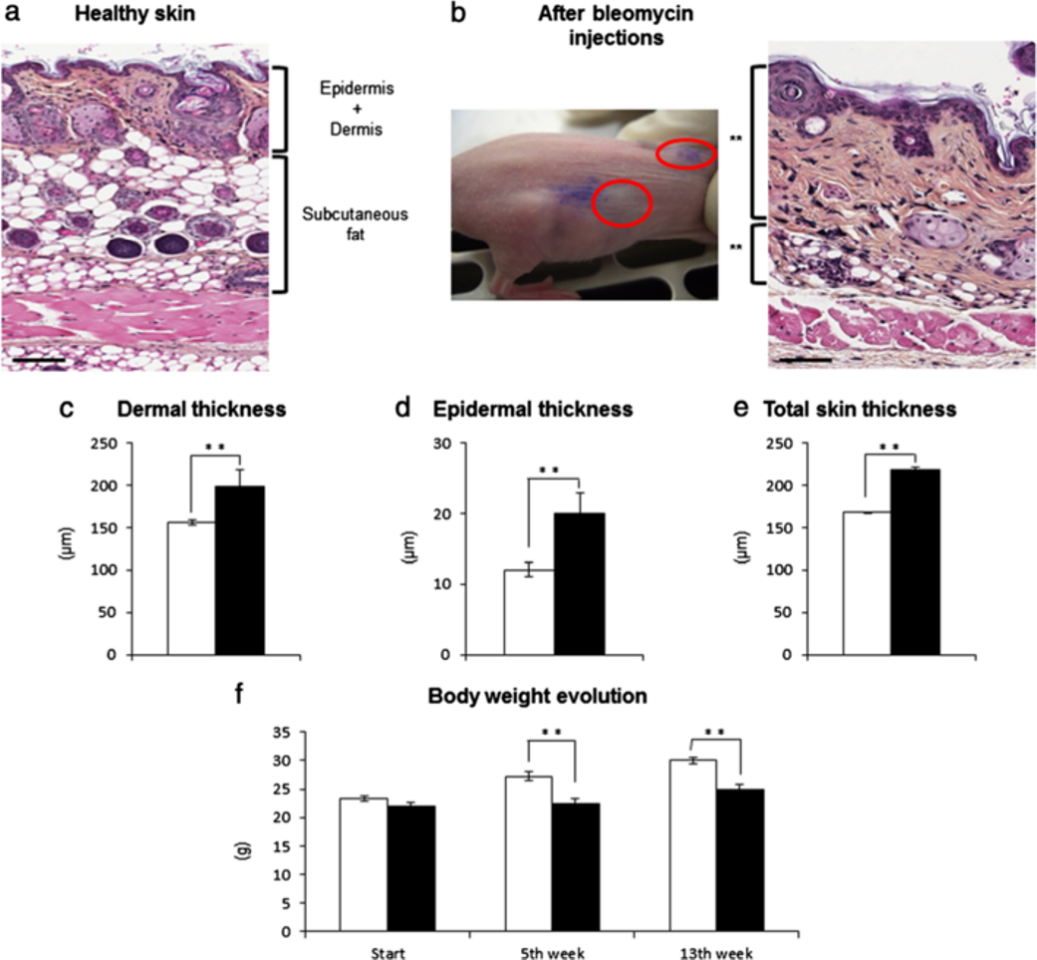
**Skin-induced sclerosis after bleomycin treatment.** Because of their inherent fragility, nude mice were injected subcutaneously with 30 mg bleomycin (BLM)/site daily during 1 month (both flanks and neck). Mice injected with 0.9% sodium chloride (NaCl) saline solution presented the same characteristics as untreated mice; only the control mice are represented. **(a)** Healthy skin (hematoxylin and eosin). **(b)** Fibrotic skin changes observed after BLM treatment. Macroscopically, injection sites appeared thicker and indurated to the touch (macroscopic picture, red ellipses). Histological analyses of BLM injection sites showed a global increase in skin thickness, and a decrease in the amount of subcutaneous fat tissue (histological picture). Comparison of **(c)** the dermis, **(d)** the epidermis and **(e)** the total skin thickness at the end of the BLM treatment (5 weeks). Data presented as mean ± standard deviation (**P <0.01, control mice *n* = 6, BLM-treated mice *n* = 6). **(f)** Time course of body weight (grams) (**P <0.01, control mice + NaCl-treated mice *n* = 24, BLM-treated mice *n* = 48). A break in the growth curve was observed at the end of BLM treatment (fifth week) and during all of the experiment (13th week). Bars = 250 μm. White, BLM-untreated mice; black, BLM-treated mice.

Repetitive sodium chloride injections did not induce skin sclerosis (data not shown). Dermis and epidermis after injection had the same thickness as untreated mice; skin of both flanks and the interscapular region also had the same thickness (mean dermal thickness = 156 ± 3 μm; mean epidermal thickness = 12 ± 1 μm; mean total skin thickness = 168 ± 3 μm; *n* = 12). Epidermis was around one-tenth of dermal thickness. Only untreated and BLM-treated mice are represented in Figure 1.

On the contrary, daily BLM subcutaneous injections during 1 month induced stable skin sclerosis (evaluated at 5 and 13 weeks post BLM treatment). Macroscopically, injection sites appeared thicker and indurated to the touch (Figure 1b, macroscopic picture). After incision, the skin showed a whitish and bright aspect compatible with sclerosis. Histopathological examination (hematoxylin and eosin) showed that all BLM-treated sites developed a stable sclerosis (mean dermal sclerosis = 198 ± 20 μm; mean epidermal sclerosis = 20 ± 3 μm; mean total skin sclerosis = 218 ± 20 μm; *n* = 12, *P* <0.01) (Figure 1c,d,e) compared with healthy skin (Figure 1a). Sclerosis is characterized by a global and homogeneous increase in the total skin thickness (the ratio epidermal/dermal sclerosis was one-tenth), a decrease in the amount of subcutaneous fat tissue (Figure 1b, histological picture), and a deposition of homogeneous materials in the thickened dermis. Masson’s trichrome and orcein stainings revealed a dense collagen network, and elastic fibers appeared more abundant and dense in the thickened dermis in BLM-treated skin than in control skin (data not shown). Toluidine blue staining revealed that there was no difference in mast cell number or morphology after BLM treatment (data not shown). These were the same histopathological features reported previously for skin fibrosis [27, 37]. Mononuclear cells were rare or absent in all analyzed skin, which was expected in nude mice.

At the end of BLM treatment, the body weights of the BLM-treated mice (fifth week; that is, 14-week-old mice) were significantly decreased (Figure 1f) compared with untreated mice. Cytotoxic effects of BLM may contribute to the break in the growth curve. The time course of body weight loss continued during the experiment (13th week; that is, 22-week-old mice) but did not affect the nude mice viability.

### Characterization and injection of the different cell therapy products

#### Stromal vascular fraction

From 220 cm^3^ harvested fat, we obtained 5.2 cm^3^ SVF with 11,530,000 cells/cm^3^, corresponding to 131,000 cells injected/mouse (0.0114 cm^3^).

#### Platelet-rich plasma

From 63 cm^3^ blood, we obtained 8.5 cm^3^ PRP with a platelet concentration of 512 G/l and 8.5 cm^3^ PPP. PRP was diluted into PPP for injections, (0.25 + 0.25) 1.28 G/l or 0.64 millions of platelets injected/mouse. Table 2 presents the PRP formulation. This formulation was similar to that previously reported for PRP purification [42, 43].

**Table 2:** **Platelet-rich plasma formulation**

Blood cells	Concentration	Cell number/mouse
Platelets	512 G/l	1,280,000,000
Red blood cells	0.13 T/l	325,000,000
White blood cells	0.88 G/l	220,000
Lymphocytes	0,75 G/l	187,500
Monocytes	0.07 G/l	17,500
Polynuclear neutrophils	0.06 G/l	15,000
Polynuclear eosinophils	0 G/l	0
Polynuclear basophils	0 G/l	0

All microbiological tests returned sterile. Composition of the different cell therapy products are presented in Table 1. Injections were made within 30 minutes after purification. Short general anesthesia and injections were well tolerated by animals.

### Clinical changes after treatment

Globally, sclerosis treated with the different cell therapy products appeared less thick and less indurated to the touch than untreated or treated sites with the appropriate dilution products, but this was generally difficult to assess. After sacrifice and during skin removal, donor fat tissue was still present in each of the three groups that included MF-derived products (that is, MF, MF + SVF and MF + PRP). MF formed homogeneous fat nodules, adherent under the skin sclerosis. We found macro-fat (Coleman’s procedure) in only one of the six mice without adhesion to the injured skin. On the contrary, we have found no trace of PRP or SVF products.

### Histopathological changes after treatment

**Figure 2:**
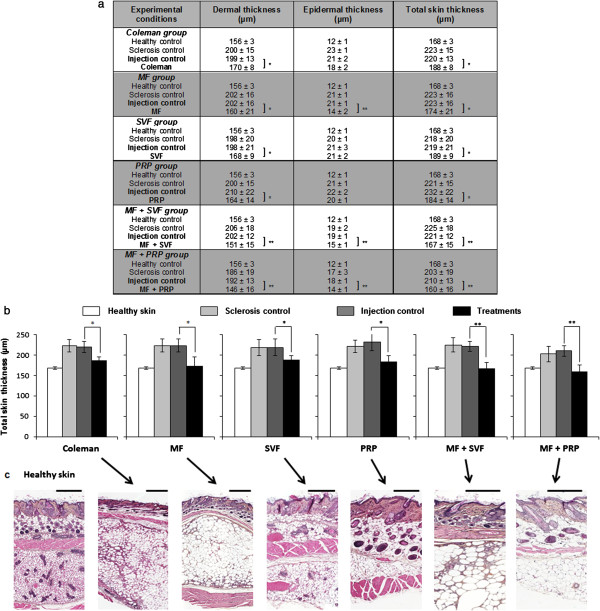
**Effects of the different cell therapy products. (a)** Dermal, epidermal and total skin thicknesses were measured (μm). Data presented as mean ± standard deviation (*n* = 6, six measures/mouse). **(b)** Graphic representation of the total skin thickness (*P <0.05, **P <0.01). White, healthy skin (no bleomycin (BLM), no treatment); grey, sclerosis control (BLM, no treatment); dark grey, injection control (BLM + sodium chloride (NaCl)); black, treatments (BLM + cell therapy products). **(c)** Corresponding histology. Bars = 500 μm. MF, micro-fat; PRP, platelet-rich plasma; SVF, stromal vascular fraction.

The injection of the appropriate dilution products (that is, Ringer’s lactate for SVF, PPP for PRP, and sodium chloride for other conditions) had no effect on sclerosis (negative control of injections checked). These injection control sites were used to compare the efficacy of the different cell therapy products on sclerosis. A significant reduction of sclerosis was reported according to the treatment. Data for dermal, epidermal, and total skin thickness for each treatment are shown in Figure 2a. With MF + SVF and MF + PRP we observed significant reduction of the dermal and the epidermal sclerosis induced by BLM treatment (*P* <0.01) (Figure 2b). Macro-fat (Coleman’s procedure), SVF and PRP only corrected dermal sclerosis (*P* <0.05). Epidermal sclerosis was reduced in treatments containing MF (*P* <0.01). In terms of collagen and elastic fibers observed in hematoxylin and eosin (Figure 2c) and Masson’s trichrome (data not shown), deposits in the dermis were still present and it was difficult to quantify and compare histologically the efficiency of the different cell therapy products. Further biochemical investigations should be conducted to better evaluate the impact of the different cell therapy products on collagen architecture and the elastic fiber density. There was no evidence of an immune response between the host animal and the grafted human material (either as rejection or graft versus host reaction).

### Fat graft viability

**Figure 3:**
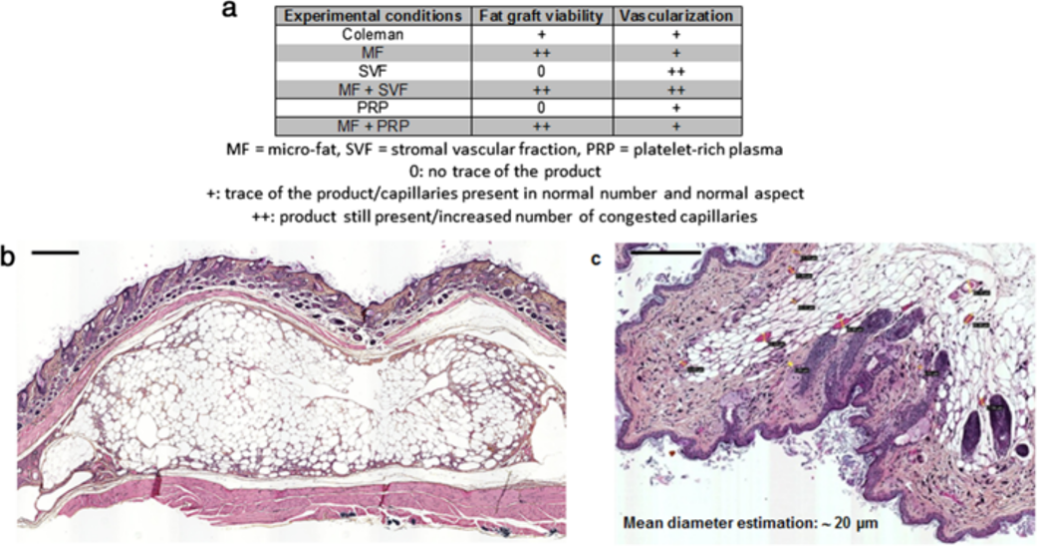
**Fat graft viability and vascularization (a).** **(b)** Example of a micro-fat (MF) nodule (dimensions 4,492 μm × 1,495 μm) still present at 2 months post injection (hematoxylin and eosin). Bars = 500 μm. Evaluation of the MF graft size suggested retention of ~6% of initial volume. **(c)** Number of congested capillaries (mean of capillary diameters = 19.6 μm), increased with stromal vascular fraction-derived products (hematoxylin and eosin, *P* <0.01). Bars = 200 μm.

MF-derived products were still present at the end of the experiment (Figure 3a); that is, 8 weeks post injections. MF formed homogeneous and adherent fat nodules under the *panniculus carnosus*. Figure 3b presents the biggest MF nodule observed. With GraphPad software, we obtained the two-dimensional dimensions: length 4,492 μm and thickness 1,495 μm. We extrapolated and calculated the approximate three-dimensional volume as around 0.03 cm^3^, which represents 6% of the injected initial volume.

### Evaluation of dermal blood vessels

Blood vessel appearance and density in areas treated with MF, PRP, or MF + PRP showed no difference from control (Figure 3a). By contrast, skin treated with either SVF alone or the combination of SVF + MF showed a significant increase of the vessel density (Figure 3a, *P* <0.01). Vessels in these groups had a congested aspect, with an estimation of their mean diameter around 19.6 μm (Figure 3c).

## Discussion

The current study demonstrated the efficacy of six different restorative biotherapies on skin-induced lesions of scleroderma. MF + SVF and MF + PRP significantly reversed dermal and epidermal sclerosis that developed after BLM treatment (*P* <0.01). Macro-fat (Coleman’s procedure), SVF, and PRP only corrected the dermal sclerosis (*P* <0.05). Epidermal sclerosis was reduced in treatments containing MF (*P* <0.01). MF-derived products were still present 8 weeks post injections. SVF products were associated with a significant increase of the local vascularization. Hence, MF-derived products were more stable and SVF demonstrated a better proangiogenic effect.

The regenerative effect appears to be associated with the duration of graft viability. Once the graft was placed in its new location, survival appeared dependent on the growth of new blood vessels in the transplanted fat fragments, resulting in a permanent correction of the treated site. In this context, the restoration of blood flow appeared essential to the survival of the graft. Grafting techniques that do not facilitate vascular development generally caused a partial or total loss of the adipose tissue graft and only obtained a short-term effect requiring repeated injections to obtain satisfactory results.

After sacrifice and during skin removal, we found no trace of PRP or SVF products, leading us to suppose that these products have been degraded. Nevertheless, PRP and SVF are known for their proangiogenic properties and they probably improve the environment of the graft, which is crucial. Vascular networks in subcutaneous tissue are too poor in their natural state to allow survival and function of the transplanted graft. Indeed, PRP or SVF cells are fragile, and even if they have proangiogenic properties, they are not able to survive over the long term. On the contrary, we observed that products containing MF were still present 8 weeks post injections. MF seems to offer favorable conditions for grafting and survival of associated PRP or SVF cells. Moreover, the number of vessels observed in the dermis was significantly increased in the SVF or MF + SVF groups, compared with the others. MF offers ideal conditions for SVF cell survival and thus may improve vascular disorders encountered in scleroderma. These data are similar to the work by Fumimoto and colleagues reporting that the creation of a rich subcutaneous vascular network with implanted adipose tissue-derived stromal cells and adipose tissue enhances subcutaneous grafting of islets in diabetic mice [44]. Hence, MF and SVF could provide superior benefits over the long term compared with either agent alone. SVF should normalize the balance of local profibrotic and antifibrotic factors and increase the microvascular network, thereby improving the skin-induced lesions of scleroderma, as suggested by the observations in this study.

It is hypothesized that when PRP is mixed with MF, the MF immediately activates the PRP, causing platelet degranulation and the release of cytokines and growth factors. This may activate MF stem cells leading to regenerative properties, and thereby allow repair of the skin-induced lesions of scleroderma. In addition, it appears that short-term effects cannot reverse epidermal lesions, and that long-term treatment may be required to reverse epidermal lesions. The final consideration for graft survival is the immune system response. To bypass the problem of histocompatibility, we used nude immunodeficient mice. Extrapolation to human studies is therefore limited.

Several open issues remain unanswered. Optimal dosing is unclear and minimal data have been published in this area. Another issue inherent to preparation of PRP products is that there are many techniques available, in terms of preparation (centrifugation speed, use of anticoagulant) and content (platelets, leukocytes, growth factors) for PRP applications. All these aspects are discussed in the literature but there is no consensus on the optimal approach. To add to the complexity, PRP content varies from one individual to another and within the same individual over the time, leading to contrasting *in vivo* effects. For this reason, the development of autologous formulations with a controlled platelet count, and consequently a controlled bioactive factor release, appears necessary [43]. In the present study, we injected 0.64 million platelets and 131,000 cells from the SVF per mouse. Evaluation of different doses to determine the optimal PRP formulation to use *in vivo* is warranted.

These data represent preliminary results. Biochemical assays (collagen, growth factors), mass spectrometry, immunological assays (cytokines) and proliferative tests of fibroblasts would be a logical next step to better understand the mechanisms involved in the observed tissue regeneration.

Separately, PRP and SVF have short-term effects, implying that repeated injections would be necessary to obtain satisfactory results. MF and derived products are capable of long-term viability, limiting the number of injections. We highlighted the interest of MF + SVF and MF + PRP mixtures compared with the MF, SVF and PRP separately for treating skin-induced lesions of scleroderma. Based on the study results, we cannot discriminate the efficacy of MF + SVF compared with MF + PRP. The only difference is that MF + SVF treatment contains more blood vessels than MF + PRP, suggesting a superior proangiogenic property and consequently a superior environment for graft survival. The use of these different regenerative products was the potential for a viable option to improve the treatment of soft tissue defects, such as scleroderma observed in human patients.

At present, there is no consensus regarding treatment for skin lesions of scleroderma. Mild forms are treated with corticosteroids for a short period (to limit the side effects associated with prolonged corticosteroid therapy). Therapeutic abstention may also be an alternative, given the lack of evidence of the effectiveness of a particular treatment. Immunosuppressive medications are considered for severe or systemic forms, but have very limited efficiency on the skin lesions. No antifibrotic treatment is known to improve skin fibrosis in this disabling disease, and thus far there is no drug that specifically reverses the microvascular injury or acts on both components together.

Fat grafting has proven to be efficient and well tolerated in the treatment of localized forms of scleroderma such as *en coup de sabre* scleroderma [45], but it has never been used to treat scleroderma skin lesions. Hand lesions are also a major problem in this disease, involving phenomena of vascular origin (ischemia), fibrotic skin lesions (scarring skin of the fingers and backs of hands) and subcutaneous calcinosis. This symptomatology has major functional consequences (pain, loss of function) and integumentary (sclerodactyly, ulcer, infection and gangrene). For scleroderma involving the hands, the volumizing effect is not desired and only the trophic effect is necessary to justify the injection of autologous SVF. The SVF is a pure condensed form of multipotent stem cells, and combined with their mechanical properties (fluidity, small amount) the cells appear suitable for treatment of fingers. The current results provide a rationale for a clinical study of fat-derived products for patients with scleroderma skin lesions, particularly those involving the hands.

## Conclusions

The current study demonstrates the efficacy of six different restorative biotherapies on skin-induced lesions of scleroderma. MF + SVF and MF + PRP significantly reversed dermal and epidermal sclerosis developed after 1 month of BLM treatment (*P* <0.01). Macro-fat (Coleman’s procedure), SVF, and PRP only corrected the dermal sclerosis (*P* <0.05). Epidermal sclerosis was reduced in treatments containing MF (*P* <0.01). MF-derived products were still present 8 weeks post injections. SVF products are associated with a significant increase in the local vascularization. Hence, MF-derived products are more stable and SVF demonstrated a better proangiogenic effect. The observed efficiency of these combination products in the animal model provides a rationale for potential clinical applications to treat human disease.

## Abbreviations

BLM: bleomycin
MF: micro-fat
PPP: platelet-poor plasma
PRP: platelet-rich plasma
SVF: stromal vascular fraction.
